# The Role of Postoperative Radiotherapy for Carcinosarcoma of the Uterus

**DOI:** 10.3390/cancers12123573

**Published:** 2020-11-30

**Authors:** Dirk Vordermark, Daniel Medenwald, Victor Izaguirre, Frank Sieker, Simone Marnitz

**Affiliations:** 1 Department of Radiation Oncology, Martin Luther University Halle-Wittenberg, 06120 Halle/Saale, Germany; daniel.medenwald@uk-halle.de (D.M.); victor.izaguirre@uk-halle.de (V.I.); frank.sieker@uk-halle.de (F.S.); 2 Department of Radiation Oncology, CyberKnife and Radiotherapy, Medical Faculty University Cologne, 50937 Cologne, Germany; simone.marnitz-schulze@uk-koeln.de

**Keywords:** carcinosarcoma, uterus, radiotherapy, brachytherapy

## Abstract

**Simple Summary:**

The role of radiotherapy on carcinosarcoma, a rare malignant tumor, of the uterus is unclear. We reviewed data published from 2010 on the effects of radiotherapy on tumor control and survival in this patient group. Available data were mainly from cancer registries and suggested that radiotherapy, given either as vaginal brachytherapy (contact radiotherapy of the vagina) or external-beam radiotherapy or a combination of both, reduces the risk of recurrence and improves survival in patients with all stages of carcinosarcoma of the uterus without metastases in other organs.

**Abstract:**

The role of postoperative radiotherapy delivered as external-beam radiotherapy (EBRT), vaginal brachytherapy (VBT) or a combination of both, in the management of carcinosarcoma of the uterus is not clearly defined, as only limited randomized trial data are available, indicating a reduction in locoregional recurrences after EBRT. We performed a structured review of data published from 2010. Although no relevant new data from prospective trials or meta-analyses were identified, 14 analyses of cancer registry data from the United States or Europe, focusing predominantly on the endpoint for overall survival, were identified, four of them using propensity-score matching to compare subgroups treated with vs. without radiotherapy. Although stage-by-stage data are rare, the registry analyses support the idea of a beneficial effect, especially of VBT, on overall survival in International Federation of Gynecology and Obstetrics (FIGO) stage IA patients (to a lesser extent in stage IB). For stages II to III, the data sets indicate the largest effects on overall survival for the combination of EBRT and VBT. In all stages, survival effects of radiotherapy apparently persist when given in addition to chemotherapy. Whereas some studies see the strongest survival effects in patients with positive lymph nodes, propensity-score matched data indicate an overall survival effect of radiotherapy (EBRT + VBT or VBT alone) in FIGO stages I to III regardless of lymph node surgery.

## 1. Introduction

Carcinosarcoma is a rare malignant tumor of the uterus containing carcinomatous and sarcomatous elements. It is considered a dedifferentiated carcinoma and accounts for approximately 5% of malignant uterine tumors. Histopathology permits the distinction of homologous uterine carcinosarcoma, containing homologous mesenchymal components derived from elements of tissue observed in the normal uterus (e.g., fibrosarcoma, leiomyosarcoma), and heterologous uterine carcinosarcoma consisting of elements derived from tissues foreign to the uterus (e.g., rhabdomyosarcoma, chondrosarcoma) [1]. For all tumors except International Federation of Gynecology and Obstetrics (FIGO) stage IV disease tumors, surgery is recommended for the purpose of staging and treatment (review in [1]).

The role of postoperative radiotherapy (RT), delivered either as external-beam radiotherapy (EBRT), vaginal brachytherapy (VBT) or a combination of both is a matter of continuing debate. The National Comprehensive Cancer Network (NCCN) Clinical Practice Guidelines “Uterine Neoplasms” Version 1.2021 gives the following recommendations for adjuvant treatment of carcinosarcoma patients following total hysterectomy with bilateral salpingo-oophorectomy and surgical staging [2]: for FIGO stage IA, systemic therapy plus VBT is the preferred option; other options are: systemic therapy with EBRT with or without VBT; VBT alone in select cases of noninvasive disease. For FIGO stages IB, II, III and IV, the advice is to give systemic therapy with or without EBRT and with or without VBT.

The recent German evidence-based guideline [3] recommends to give postoperative radiotherapy to improve local control in carcinosarcoma of FIGO stages I and II. In the same guideline, adjuvant chemotherapy with cisplatin and ifosfamide (carboplatin/paclitaxel as alternative) is recommended as optional in stages I and II, whereas for stages III and IV a survival benefit of the cisplatin/ifosfamide combination (as compared to ifosfamide montotherapy) is described in an evidence-based statement [3].

Although the guideline recommendations address both the locoregional and distant aggressiveness of carcinosarcoma, with 10% distant metastasis and 60% extrauterine growth at diagnosis [1], there is a need to more clearly define the role of and the potential benefit from postoperative radiotherapy, in particular of both its relevant modalities EBRT and VBT. This should address the different FIGO stage groups of uterine carcinosarcoma and the relation to other treatment modalities, especially the extent of surgery and the use of chemotherapy.

Previous recommendations were predominantly founded on limited randomized trial data and knowledge from analyses of cancer registry data published up until about the year 2010. As carcinosarcoma was excluded from major randomized trials in endometrial cancer, such as the PORTEC trials, the highest-level evidence came from the randomized European Organization for the Research and Treatment of Cancer (EORTC) Trial 55,874 [4]. Among 224 patients with different histologies of uterine sarcomas, 92 patients with carcinosarcoma of FIGO stages I or II were randomized to receive EBRT with 50.4 Gy in fractions of 1.8 Gy vs. observation. After a median follow-up of 6.8 years, a significant reduction in the local recurrence rate from 47% to 24% was observed. A beneficial effect on survival was also described but this was not significant with a hazard ratio (HR) of 1.58 (95% CI = 0.83–3.01). The concept of postoperative whole-abdominal irradiation with a boost to the pelvis was compared with combination chemotherapy in 206 patients (45% FIGO stage III) in the randomized Gynecologic Oncology Group (GOG) Trial 150 [5]. No improvements in overall survival or recurrence rates were seen for the large-volume (but limited-dose) radiotherapy concept.

Analyses of United States cancer registry databases published up until 2010 predominantly showed a positive effect of adjuvant pelvic EBRT (with or without VBT) on locoregional recurrence-free survival and overall survival in uterine carcinosarcoma [6,7,8], although some authors have discussed that the effect of radiotherapy on survival may be related to the performance and extent of lymphadenectomy [9].

Since 2010, the patterns of treatment of uterine carcinosarcoma may have changed and new data sets have emerged, including population-based registry data analyzed with alternative statistical methods such as propensity-score matching [10]. To more clearly define the role of postoperative radiotherapy for uterine carcinosarcoma, we performed a structured review of publications since the year 2010.

## 2. Results

Fourteen publications fulfilled the defined criteria and were analyzed further as described below.

### 2.1. Prospective Trials

Only one prospective trial, the SARCGYN study from the French Sarcoma Group, was identified [11]. A group of 81 patients with different histologies of uterine sarcoma, including 19 cases of carcinosarcoma (FIGO stages I to III), were randomized to receive adjuvant pelvic EBRT with 45 Gy vs. the same radiotherapy plus chemotherapy with doxorubicin, ifosfamide and cisplatin. While the addition of chemotherapy significantly improved 3-year disease-free survival for the mixed-histology overall group from 69% to 81% (*p* = 0.041); no specific data for the subgroup of carcinosarcoma were reported.

### 2.2. Meta-Analyses

One meta-analysis was identified—a Cochrane systematic review on adjuvant radiotherapy and/or chemotherapy after surgery for carcinoma published first in 2011 and then as an update in 2013 [12,13]. The meta-analysis included three randomized clinical trials, two of which evaluated multiagent vs. single-agent chemotherapy, the third being the above-mentioned trial GOG 150 [5], showing no significant difference in overall or recurrence-free survival between adjuvant chemotherapy with three cycles of cisplatin, ifosfamide and mesna vs. adjuvant radiotherapy including whole-abdominal irradiation. Thus, no additional information on the value of radiotherapy in this setting could be gained from the meta-analyses.

### 2.3. Cancer Registry Data

Nine publications reported on analyses of cancer registry data sets from the United States, in particular the Surveillance, Epidemiology and End Results (SEER) database, a set of population-based registry data covering approximately 28% of the United States population and the National Cancer Database (NCDB), a hospital-based registry capturing about 70% of incident cancer cases in the United States. In these analyses, different time periods and inclusion criteria as well as varying statistical methods were applied. A summary of these data sets is presented in Table 1 for SEER and in Table 2 for NCDB.

Three reports from European Cancer Registries were found—two from the national Netherlands Cancer Registry and one from a regional registry in Sweden. The main results are summarized in Table 3.

## 3. Discussion

To identify new data on the benefit from postoperative radiotherapy, delivered as EBRT, VBT or a combination of both, for uterine carcinosarcoma, a structured analysis of new findings published from 2010 was performed. Our search retrieved no relevant new knowledge from prospective or even randomized trials or from meta-analyses. However, 14 reports on analyses of the effects of postoperative RT for carcinosarcoma in national or regional cancer registry data sets were identified, mostly from the United States databases SEER and NCDB. Although such sources cannot replace data from randomized clinical trials, some of the recent reports utilized advanced statistical methods, in particular propensity-score matching, to address imbalances inherent to retrospectively collected data. This discussion will now focus on the data sets where propensity matching was applied.

In their report from the SEER database (time period 2004–2013, FIGO stages I–III), Li et al. found in their matched cohort analysis significant and clinically relevant effects of EBRT, of VBT and of their combination on overall survival, with hazard ratios of 0.65 and 0.62 for the single radiotherapy modalities and 0.47 for the combination [15]. Specific data for individual FIGO stages were not presented. Any radiotherapy was only used in 40% of all carcinosarcoma cases included in this report, leading the authors to conclude that radiotherapy has been underused in carcinosarcoma.

Odei et al. investigated the effect of adding radiotherapy (EBRT + VBT or VBT alone) to chemotherapy (stages I–IV, SEER, 2004–2012) and found in the matched analysis that providing radiotherapy to this patient group significantly improved overall survival with a hazard ratio of 0.68 [18]. In the Forrest plot, this effect was maintained in almost all clinical subgroups, significantly so in FIGO stages I, II and III and in both patients with and without lymph node surgery. Distinct effects of the radiotherapy modalities, EBRT + VBT as compared to VBT alone, were not analyzed.

Stokes et al. found in their matched analysis of NCDB data (2004–2012, stages I–III) an improvement of overall survival by the combination of EBRT + VBT with a hazard ratio of 0.74 [19]. EBRT alone and VBT alone also had a tendency to decrease the risk with hazard ratios of 0.89 and 0.80, respectively, but these effects were not significant. This report contained data on specific FIGO stages, but only from the unmatched analysis where the only significant effect on overall survival was an improvement with VBT in FIGO II patients.

Finally, Seagle et al. specifically investigated stage I (NCDB, 1998–2013) and found after propensity-score matching that VBT was associated with improved overall survival in the whole group (hazard ratio 0.83) and even stronger in the group with pathologically negative lymph nodes (HR 0.80) [21].

What do these and other findings of multivariate analyses—but without propensity-score matching—add to current knowledge on the indication for postoperative radiotherapy in carcinosarcoma? The best evidence so far has come from a group of 92 carcinosarcoma patients (FIGO stages I and II) randomized to EBRT with 50.4 vs. observation in the EORTC 55,874 Trial [4]. Over the recruitment period from 1988 to 2001, none of these patients received a lymphadenectomy (lymph node sampling was recommended but performed in only about 25% across all histologies included in this trial) and no patient received chemotherapy. The primary endpoint was locoregional recurrence including local (vaginal and paravaginal) and regional (other pelvic) sites of recurrence. After a median follow-up of 6.8 years, locoregional recurrence occurred in 24% of patients after radiotherapy and in 47% after observation. Although no significant effect on survival was seen, the tendency was reported as “beneficial” for the radiotherapy group (HR 1.58, 0.83–3.01). The trial was powered to analyze effects in the whole group of various histologies and not planned to specifically investigate carcinosarcoma.

A question arises concerning if the effect seen in EORTC 55,874 on locoregional control with EBRT (and potentially a positive effect on survival) can also be achieved with VBT—with less toxicity—as has been shown for endotrial adenocarcinoma in the PORTEC-2 trial [26]. For the group of FIGO IA patients, the multivariate analysis (although without matching) by Shinde et al. suggests that this most favorable subgroup has an overall survival benefit from VBT, but not from EBRT [20].

For the remaining stage I patients, i.e., stage IB, the most relevant data may be from Seagle et al., showing a significant improvement of overall survival with VBT, but only a trend for EBRT. Thus in the registry data sets focusing on stage, no general preference over EBRT can be derived. Unfortunately, the recommendation of RT strategies for clinically relevant combinations of factors, including FIGO stage as well as other risk factors, cannot easily be derived from these data.

A significant impact of EBRT on overall survival becomes more prominent in the studies of patient groups including FIGO stages higher than I. Although these mostly do not contain stage-by-stage analyses, pronounced benefits, especially of the combination of EBRT and VBT, have been seen in the stage groups I-III by Li et al., with a hazard ratio of 0.47 [15], and by Stokes et al., with a hazard ratio of 0.74 [19], with both effects being stronger than those of EBRT of VBT alone in these studies, suggesting that patients at stage II or III benefit from radiotherapy consisting of more than VBT alone, possibly the combination of EBRT and VBT.

The data from Odei et al. [18] suggest that overall survival effects from radiotherapy (of either EBRT + VBT or VBT alone) persist when radiotherapy is given in addition to chemotherapy and that this effect is present through FIGO stages I, II and III as well as in both patients with and without lymph node surgery. The Dutch registry data support the benefit of adding EBRT to chemotherapy specifically for stage III [23]. A limitation of these data sets is that the specific chemotherapy protocols used were not available to the authors of these reports [18,23].

Any effects of radiotherapy on locoregional recurrence rates, as found in the randomized EORTC 55,874 Trial, cannot be evaluated in the NCDB and SEER databases as these report overall survival only. However, where significant effects of radiotherapy on overall survival are seen it must be assumed that these result from rather pronounced reductions in (nonsalvagable) locoregional recurrences as shown in the randomized trial. Although smaller in size, the Swedish regional registry data set by Sorbe et al. supports a halving of locoregional recurrences by EBRT in stages I and II carcinosarcoma (8% vs. 19%) and an additional effect, here on relapse-free survival, of adding EBRT (±VBT) to chemotherapy in stages III to IV [25].

## 4. Materials and Methods

The database Pubmed was searched with the following search strategy for the time period from 1 January 2010 to 20 October 2020: (carcinosarcoma AND (uterus OR uterine) AND (radiotherapy OR brachytherapy)). A number of 138 abstracts were identified and reviewed. Only reports of prospective trials, meta-analyses and national or regional cancer registry analyses were considered. Single-institution or multi-institutional retrospective case series were excluded.

## 5. Conclusions

In conclusion, in the absence of additional prospective trial data, new registry data indicate overall survival benefits of radiotherapy in patients with FIGO stage I to III carcinosarcoma which persist when radiotherapy is added to chemotherapy and may be independent of lymphadenectomy. The survival impact appears to be strongest for vaginal brachytherapy in stage IA (possibly less in IB) and for a combination of external-beam radiotherapy and brachytherapy in stages II and III. Recommendations derived from our analysis are summarized in Table 4.

## Figures and Tables

**Table 1:** Overview of studies of postoperative radiotherapy in uterine carcinosarcoma using data from the United States Surveillance, Epidemiology and End Results (SEER) database (2010–2020).

Authors	Data Source (Time Period)	Inclusion Criteria	*n*	Main Result	Comment
Nama et al., 2020 [14]	SEER (1973–2010)	stages I–IV	3706	RT independently associated with survival (OR 0.1, CI = 0.02–0.06, *p* < 0.005)	no specific data by stage or by type of RT (EBRT, VBT)
Li et al., 2019 [15]	SEER (2004–2013)	stages I–III	1069	multivariate Cox analysis: overall survival benefit for EBRT HR 0.72 (0.53–0.99; *p* = 0.042), VBT HR 0.55 (0.37–0.80; *p* = 0.002), combination HR 0.47 (0.29–0.77; *p* = 0.003) propensity-score matched: overall survival benefit EBRT HR 0.65 (0.45–0.93; *p* = 0.02), VBT 0.62 (0.40–0.95; *p* = 0.029), combination HR 0.47 (0.26–0.85; *p* = 0.013)	
Manzerova et al., 2016 [16]	SEER (1999–2010)	stages I–IV	2342	EBRT effect in most recent time period 2005–2010: crude 5-year OS with EBRT 50.9%, without 33% (*p* < 0.0001); median OS stage I with RT 71 mo vs. without EBRT 43 mo; stage II 52 vs. 18 mo.; stage III 39 vs. 29 mo (all *p* ≤ 0.007), stage IV 14 vs. 9 mo (*p* < 0.10)	VBT not studied: OS effects of EBRT remain after correction for prognostic factors
Patel et al., 2015 [17]	SEER (1998–2010)	stages I–II	1581	multivariate Cox model: OS improved by VBT vs. no RT HR 0.67 (CI 0.47–0.95; *p* = 0.024), EBRT vs. no RT HR 0.90 (0.76–1.06; *p* = 0.22)	authors conclude EBRT not better than VBT
Odei et al., 2018 [18]	SEER (2004–2012)	stages I–IV receiving chemotherapy (±RT)	3538	multivariate analysis: OS benefit from addition of RT to chemotherapy, HR 0.67 (0.55–0.81); no significant difference OS between EBRT and VBT; propensity-score matched: OS improvement by addition of RT, HR 0.68 (0.61–0.77; *p* < 0.01)	RT was either EBRT + VBT or VBT alone, OS benefit from RT in most subgroups (propensity-score matched), including FIGO stages I–III and with/without lymph node surgery

**Table 2:** Overview of studies of postoperative radiotherapy in uterine carcinosarcoma using data from the United States National Cancer Database (NCDB) (2010–2020).

Authors	Data Source (Time Period)	Inclusion Criteria	*n*	Main Result	Comment
Stokes et al., 2018 [19]	NCDB (2004–2012)	stages I–III	2303	multivariate analysis: overall survival improved by combination EBRT + VBT, HR 0.72 [0.58–0.89; *p* < 0.01), not by EBRT alone, HR 0.93 (0.79–1.10; *p* = 0.41) or VBT alone, HR 0.84 (0.68–1.02; *p* = 0.09); propensity-score-matched analysis: overall survival improved by combination EBRT + VBT, HR 0.74 (0.58–0.96; *p* = 0.02), not by EBRT alone, HR 0.89 (0.73–1.07; *p* = 0.34) or by VBT alone, HR 0.80 (0.63–1.03; *p* = 0.09)	data for individual FIGO stages only in unmatched groups: OS advantage of VBT in stage II
Shinde et al., 2018 [20]	NCDB (2004–2015)	stage IA	2701	multivariate analysis: OS benefit from VBT, HR 0.80, *p* = 0.047; not from EBRT, HR 0.89, *p* = 0.28	
Seagle et al., 2017 [21]	NCDB (1998–2013)	stage I	5614	multivariate analysis: improvement of OS by EBRT, HR 0.91 (0.82–1.01; *p* = 0.08), by VBT, HR 0.81 (0.70–0.95; *p* = 0.07), by EBRT + VBT, HR 0.88 (0.73–1.06; *p* = 0.16); propensity-score-matched analysis: VBT associated with improved OS, HR 0.83 (0.70–0.97; *p* = 0.02); in subgroup with pathologically negative lymph nodes HR 0.80 (0.67–0.96; *p* = 0.01)	
Wong et al. [22]	NCDB (2004–2011)	stages I–IIIC1	4906	node-negative patients: 5-year OS with chemo + EBRT 65.2% vs. chemo + VBT 70.4% (*p* = 0.07); node-positive with chemo + EBRT 50.5% vs. chemo + VBT 31.7% (*p* = 0.07); multivariate analysis: OS benefit with chemo + RT any modality, HR 0.50 (0.44–0.57; *p* < 0.001), not from RT alone	in some analyses RT modality (EBRT vs. VBT) not differentiated, both considered as RT

**Table 3:** Overview of studies of postoperative radiotherapy in uterine carcinosarcoma using data from European cancer registries (2010–2020).

Authors	Data Source (Time Period)	Inclusion Criteria	*n*	Main Result	Comment
van Welden et al., 2020 [23]	Netherlands Cancer Registry (2005–2016)	stage III	132	combination of chemotherapy and EBRT associated with improved OS compared to chemotherapy alone, HR 2.49 (1.24–4.99; *p* = 0.01), to EBRT alone, HR 2.53 (1.29 –4.97; *p* = 0.007)	
Versluis et al., 2018 [24]	Netherlands Cancer Registry (1993–2012)	stages I–IV	1140	multivariate analysis: RT improves OS, HR 0.65 (0.55–0.77; *p* < 0.001), strongest effect from chemotherapy + RT, HR 0.25 (0.14–0.47; *p* < 0.001); in subgroup lymphadenectomy node-negative: nonsignificant OS effect of RT, HR 0.65 (0.39–1.09, *p* = 0.1) and of chemotherapy + RT, HR 0.68 (0.20–3.24; *p* = 0.45; in subgroup lymphadenectomy node-positive significant OS effect of RT, HR 0.17 (0.07–0.39; *p* < 0.001) and of chemotherapy + RT, HR 0.04 (0.03–0.18; *p* < 0.001)	modality of RT (EBRT vs. VBT) not specified
Sorbe et al., 2013 [25]	Sweden, regional registry (1973–2007)	stages I–IV	322	stages I–II crude locoregional recurrence 8% with EBRT (±VBT) vs. 19% without (*p* = 0.0103); stages III–IV multivariate analysis: improvement of relapse-free survival by addition of EBRT (±VBT) to chemotherapy, HR 0.62 (0.46–0.85)	

**Table 4:** Overview of recommendations derived in the present analysis regarding the use of postoperative external-beam radiotherapy (EBRT) and/or vaginal brachytherapy (VBT). The recommendations reflect author opinion, not an evidence-based grading system (+: recommended; 0: unclear; -: not recommended).

Stage	EBRT Alone	VBT Alone	EBRT + VBT	Comment
IA	(+)	+	(+)	best OS data for VBT alone
IB	+	+	+	no clear advantage for any modality or combination
II	(+)	(+)	+	best OS data for combination
III	(+)	(+)	+	best OS data for combination
IV	(+)	−	0	limited data for EBRT in stage IV

## References

[1] Denschlag D., Ulrich U.A. (2018). Uterine Carcinosarcomas—Diagnosis and Management. Oncol. Res. Treat..

[2] National Comprehensive Cancer Network (2021). NCCN Clinical Practice Guidelines in Oncology: Uterine Neoplasms.

[3] Emons G., Steiner E., Vordermark D., Uleer C., Bock N., Paradies K., Ortmann O., Aretz S., Mallmann P., Kurzeder C. (2018). Interdisciplinary Diagnosis, Therapy and Follow-up of Patients with Endometrial Cancer. Guideline (S3-Level, AWMF Registry Nummer 032/034-OL, April 2018)—Part 1 with Recommendations on the Epidemiology, Screening, Diagnosis and Hereditary Factors of Endometrial Cancer. Geburtshilfe Frauenheilkd..

[4] Reed N.S., Mangioni C., Malmström H., Scarfone G., Poveda A., Pecorelli S., Tateo S., Franchi M., Jobsen J.J., Coens C. (2008). European Organisation for Research and Treatment of Cancer Gynaecological Cancer Group. Phase III randomised study to evaluate the role of adjuvant pelvic radiotherapy in the treatment of uterine sarcomas stages I and II: An European Organisation for Research and Treatment of Cancer Gynaecological Cancer Group Study (protocol 55874). Eur. J. Cancer.

[5] Wolfson A.H., Brady M.F., Rocereto T., Mannel R.S., Lee Y.C., Futoran R.J., Cohn D.E., Ioffe O.B. (2007). A gynecologic oncology group randomized phase III trial of whole abdominal irradiation (WAI) vs. cisplatin-ifosfamide and mesna (CIM) as post-surgical therapy in stage I-IV carcinosarcoma (CS) of the uterus. Gynecol. Oncol..

[6] Sampath S., Schultheiss T.E., Ryu J.K., Wong J.Y. (2010). The role of adjuvant radiation in uterine sarcomas. Int. J. Radiat. Oncol. Biol. Phys..

[7] Smith D.C., Macdonald O.K., Gaffney D.K. (2008). The impact of adjuvant radiation therapy on survival in women with uterine carcinosarcoma. Radiother. Oncol..

[8] Wright J.D., Seshan V.E., Shah M., Schiff P.B., Burke W.M., Cohen C.J., Herzog T.J. (2008). The role of radiation in improving survival for early-stage carcinosarcoma and leiomyosarcoma. Am. J. Obstet. Gynecol..

[9] Nemani D., Mitra N., Guo M., Lin L. (2008). Assessing the effects of lymphadenectomy and radiation therapy in patients with uterine carcinosarcoma: A SEER analysis. Gynecol. Oncol..

[10] Biondi-Zoccai G., Romagnoli E., Agostoni P., Capodanno D., Castagno D., D’Ascenzo F., Sangiorgi G., Modena M.G. (2011). Are propensity scores really superior to standard multivariable analysis?. Contemp. Clin. Trials.

[11] Pautier P., Floquet A., Gladieff L., Bompas E., Ray-Coquard I., Piperno-Neumann S., Selle F., Guillemet C., Weber B., Largillier R. (2013). A randomized clinical trial of adjuvant chemotherapy with doxorubicin, ifosfamide, and cisplatin followed by radiotherapy versus radiotherapy alone in patients with localized uterine sarcomas (SARCGYN study). A study of the French Sarcoma Group. Ann. Oncol..

[12] Galaal K., Godfrey K., Naik R., Kucukmetin A., Bryant A. (2011). Adjuvant radiotherapy and/or chemotherapy after surgery for uterine carcinosarcoma. Cochrane Database Syst. Rev..

[13] Galaal K., van der Heijden E., Godfrey K., Naik R., Kucukmetin A., Bryant A., Das N., Lopes A.D. (2013). Adjuvant radiotherapy and/or chemotherapy after surgery for uterine carcinosarcoma. Cochrane Database Syst. Rev..

[14] Nama N., Cason F.D., Misra S., Hai S., Tucci V., Haq F., Love J., Ullah A., Peterson P., Grishko F.F. (2020). Carcinosarcoma of the Uterus: A Study From the Surveillance Epidemiology and End Result (SEER) Database. Cureus.

[15] Li Y., Ren H., Wang J. (2019). Outcome of adjuvant radiotherapy after total hysterectomy in patients with uterine leiomyosarcoma or carcinosarcoma: A SEER-based study. BMC Cancer.

[16] Manzerova J., Sison C.P., Gupta D., Holcomb K., Caputo T.A., Parashar B., Nori D., Wernicke A.G. (2016). Adjuvant radiation therapy in uterine carcinosarcoma: A population-based analysis of patient demographic and clinical characteristics, patterns of care and outcomes. Gynecol. Oncol..

[17] Patel N., Hegarty S.E., Cantrell L.A., Mishra M.V., Showalter T.N. (2015). Evaluation of brachytherapy and external beam radiation therapy for early stage, node-negative uterine carcinosarcoma. Brachytherapy.

[18] Odei B., Boothe D., Suneja G., Werner T.L., Gaffney D.K. (2018). Chemoradiation Versus Chemotherapy in Uterine Carcinosarcoma: Patterns of Care and Impact on Overall Survival. Am. J. Clin. Oncol..

[19] Stokes W.A., Jones B.L., Schefter T.E., Fisher C.M. (2018). Impact of radiotherapy modalities on outcomes in the adjuvant management of uterine carcinosarcoma: A National Cancer Database analysis. Brachytherapy.

[20] Shinde A., Li R., Amini A., Chen Y.J., Cristea M., Dellinger T., Wang W., Wakabayashi M., Beriwal S., Glaser S. (2018). Improved survival with adjuvant brachytherapy in stage IA endometrial cancer of unfavorable histology. Gynecol. Oncol..

[21] Seagle B.L., Kanis M., Kocherginsky M., Strauss J.B., Shahabi S. (2017). Stage I uterine carcinosarcoma: Matched cohort analyses for lymphadenectomy, chemotherapy, and brachytherapy. Gynecol. Oncol..

[22] Wong A.T., Lee Y.C., Schwartz D., Lee A., Shao M., Han P., Choi K., Schreiber D. (2017). Use of Adjuvant Chemotherapy, Radiation Therapy, or Combined Modality Therapy and the Impact on Survival for Uterine Carcinosarcoma Limited to the Pelvis. Int. J. Gynecol. Cancer..

[23] van Weelden W.J., Reijnen C., Eggink F.A., Boll D., Ottevanger P.B., van den Berg H.A., van der Aa M.A., Pijnenborg J.M.A. (2020). Impact of different adjuvant treatment approaches on survival in stage III endometrial cancer: A population-based study. Eur. J. Cancer.

[24] Versluis M.A.C., Pielsticker C., van der Aa M.A., de Bruyn M., Hollema H., Nijman H.W. (2018). Lymphadenectomy and Adjuvant Therapy Improve Survival with Uterine Carcinosarcoma: A Large Retrospective Cohort Study. Oncology.

[25] Sorbe B., Paulsson G., Andersson S., Steineck G. (2013). A population-based series of uterine carcinosarcomas with long-term follow-up. Acta Oncol..

[26] Wortman B.G., Creutzberg C.L., Putter H., Jürgenliemk-Schulz I.M., Jobsen J.J., Lutgens L.C.H.W., van der Steen-Banasik E.M., Mens J.W.M., Slot A., Kroese M.C.S. (2018). PORTEC Study Group. Ten-year results of the PORTEC-2 trial for high-intermediate risk endometrial carcinoma: Improving patient selection for adjuvant therapy. Br. J. Cancer.

